# Smartphone-based surface topography app accurately detects clinically significant scoliosis

**DOI:** 10.1007/s43390-025-01062-7

**Published:** 2025-04-02

**Authors:** Matthew S. Rohde, Marleni Albarran, Anthony A. Catanzano, Elizabeth J. Sachs, Hiba Naz, Amishi Jobanputra, Jacob Ribet, Kali Tileston, John S. Vorhies

**Affiliations:** 1 Stanford University School of Medicine Department of Orthopedic Surgery, 453 Quarry Rd, 3rd Floor, MC 5658, Palo Alto, CA USA; 2 Duke University School of Medicine Department of Orthopedic Surgery, Durham, NC USA

**Keywords:** Idiopathic scoliosis, Adult, Surface topography, Scoliometer, Digital health, Accuracy, 3D scanning

## Abstract

**Purpose:**

The purpose of this study was twofold: (1) to validate the predictive capabilities of the Scoliosis Assessment App using ST technology against X-ray “ground truth” in patients being evaluated for clinically significant scoliosis; and (2) to compare the diagnostic accuracy of the App versus the commonly used scoliometer tool.

**Methods:**

A multicenter, prospective validation study was conducted among patients with known or suspected scoliosis. The App determined an Asymmetry Index to predict the likelihood of clinically significant disease (MCM ≥ 20°) as determined by X-ray. Outcomes included the sensitivity, specificity, and area under the receiver operating characteristic curve (ROC AUC) associated with the Apps prediction of clinically significant disease.

**Results:**

Fifty-five patients were evaluated with a mean age of 13.6 ± 2.1 years. The App correctly classified 91% (50/55) of the patients compared to 69% (38/55) for the scoliometer. The sensitivity of the App was 96.4% (89.6–100% CI) versus 50% (28.1–71.9% CI) for the scoliometer (*P* < 0.05), while the specificity values were 85.2% (71.8–98.9% CI) and 88.9% (74.4–100% CI), respectively. ROC analysis indicated a statistically significant difference in accuracy (AUC) in favor of the App (95% versus 71%; *P* = 0.015).

**Conclusion:**

The Scoliosis Assessment App using ST technology offers an accurate, accessible, and non-ionizing method of detecting clinically significant scoliosis, suggesting that the App can be used for detection and monitoring as an alternative to radiography and as a replacement for scoliometer without diminishing the standard of care. Further studies are required to assess variations of sensitivity in a large cohort of patients and clinical utility as an alternative to radiographs.

## Introduction

Scoliosis is a common condition in the United States and globally with an estimated prevalence of 3% to 4% in adolescents and approximately 38% among adults [1, 2]. Initial diagnosis and stratification within treatment algorithms hinges upon measurements made on plain radiographs, [3] but evaluation is typically performed by clinical exam often supplemented with scoliometer. The scoliometer is a type of inclinometer which itself has limited sensitivity and specificity that can lead to late detection and inappropriate referrals [4]. Inaccurate evaluation can result increased morbidity for patients as well as incur substantial healthcare costs because of unnecessary specialist referrals and increased likelihood of surgical intervention [3–6]. X-ray evaluation is accurate but is undesirable given the associated costs and exposure to ionizing radiation. For these reasons a scalable, accurate radiation-free method for diagnosing and monitoring scoliosis could offer significant benefit to patients and the healthcare system.

While radiation doses are relatively low for follow-up plain X-rays, the cumulative effects of radiation exposure can confer an increased risk of malignancy [7]. Various methods of 3D analysis of trunk surface topography (ST) have shown promise as an effective, radiation-free tool to evaluate patients for scoliosis [8–10]. The results of these studies proposed ST as a method of longitudinal assessment of deformity, although it did accurately estimate Cobb angle (major curve magnitude) [9]. However, most of the described methods for analyzing ST are not scalable given the need for costly and cumbersome equipment. This has limited the incorporation of ST-based assessment into clinical practice.

The Scoliosis Assessment App (NSite Medical, Inc, Menlo Park, California, USA) leverages the optical sensors embedded in a smartphone to calculate the probability that a patient has clinically significant scoliosis based on a 3D scan of the torso. Of note, both the Scoliosis Assessment App and X-ray measure the same aspect of the deformity, the ascertainment of major curve magnitude (MCM). The probabilities generated by the Scoliosis Assessment App are intended to be used as a clinical decision support tool and has the potential to facilitate scoliosis diagnosis and monitoring by healthcare providers. This study aimed (1) to validate the ability of the Scoliosis Assessment App using ST technology to predict clinically significant scoliosis as defined by conventional radiographic measurements; and (2) to compare the diagnostic accuracy of the App to the commonly used scoliometer tool.

## Methods

After Institutional Review Board approval, a multicenter, prospective validation study was conducted among patients being evaluated for known or suspected scoliosis at two academic quaternary care pediatric healthcare systems in distinct geographic regions from August 8, 2023 to October 3, 2023. Inclusion criteria were patients aged 10 to 18 years old that had an anterior–posterior or posterior-anterior scoliosis radiograph within 30 days of enrollment. Exclusion criteria were non-ambulatory, diagnosis of a syndrome or neuromuscular condition associated with scoliosis, and any history of spine or chest wall fracture or surgery. The device received 510(k) clearance from the United States Food and Drug Administration on November 15, 2023 (ClinicalTrials.gov NCT06035952) [11].

### Data collection

After informed consent and assent was obtained, Scoliosis Assessment App scans were performed during the clinic visit. Scans were performed by clinical research coordinators trained on the use of the App by reviewing the user manual and completing three practice scans under the supervision of a trained scanner. Patients were prepared with pants below the posterior superior iliac spine, hair held aside so the neck was visible up to the hairline, and upper body undressed of loose-fitting clothing. The 3D images were obtained with the patient bent forward over with arms hanging perpendicular to the ground, palms facing together, and knees extended with feet together in the position of Adam’s forward bend test. Participants underwent a scoliometer assessment of angle of trunk rotation (ATR) by trained research coordinators during the same visit. The assessor stood behind the patient and assessed the horizontal plane of the spine, placing the half-circle cutout of the scoliometer over the top of the spinous process and measuring the maximal ATR on the thoracic and lumbar spine. The height of the patient’s bending position was adjusted as needed so that the deformity of the spine was most pronounced to find the maximum thoracic or lumbar ATR. Coordinators obtaining the ATR were blinded to the 3D and radiographic measurements. For the purposes of obtaining inter- and intra-rater reliability, ten consecutive patients had scans obtained from three separate users and ten patients had two separate scans taken by the same user on the same day.

Major coronal curve magnitude was measured by an attending pediatric orthopedic surgeon or trained research coordinator who was blinded to the results of the 3D scan using Sectra radiographic imaging software (Sectra; Linköping, Sweden). Demographic data, including age, gender, race, ethnicity, and BMI, were recorded through chart review of each patient.

### Statistical measures

**Fig. 1:**
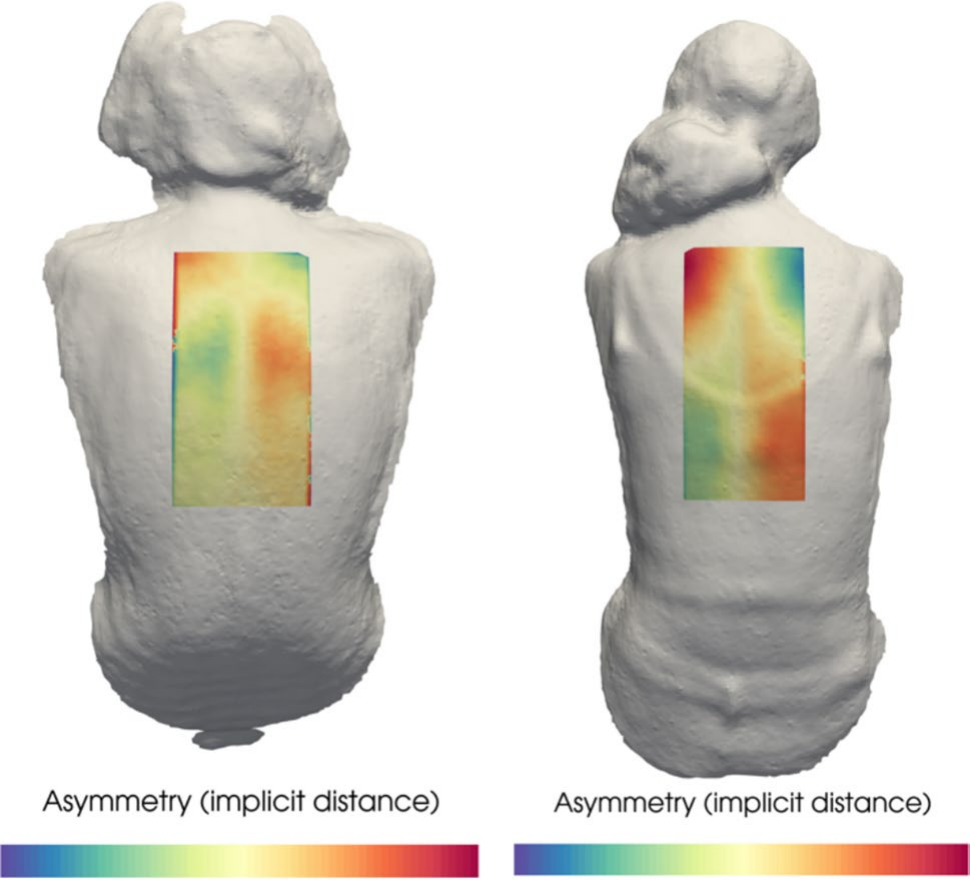
Applied heat map for calculation of Scoliosis Assessment App Asymmetry Index. Patient on left with lumbar MCM of 5°, scoliometer of 7 and Asymmetry Index 0.93 (31.9% predicated probability of clinically significant scoliosis) compared to patient on right with thoracic curve MCM 23°, scoliometer of 11, and Asymmetry Index of 2.91 (58.8% predicted probability of clinically significant scoliosis)

The deidentified scans were sent to secure cloud-based servers and were processed by personnel blinded to the clinical data collected. Scans were placed into standard alignment based on ordinal planes using a 3D software package (Meshlab) and a region of interest was defined for lumbar or thoracic curves on the back surface for the purpose of quantifying topographical asymmetry (Fig. 1**)**. To determine the reproducibility of the manual alignment of the scans, four personnel aligned each scan after familiarizing themselves with the user manual. The software then calculated an Asymmetry Index based on a previously described method [8]. This Asymmetry Index was then used to generate a predicted probability of the patient having a coronal MCM based on relationships between ST and radiographic measurements previously established in App development and pilot testing.

Descriptive statistics were generated to illustrate the distributions of population demographics and curve type and severity. Diagnostic ability (accuracy) of Asymmetry Index versus scoliometer ATR was assessed through calculations of area under the receiver operating characteristic curve (ROC AUC). For the purposes of sensitivity and specificity calculations, a predictive probability threshold of 50% likelihood of coronal MCM ≥ 20° was defined as a true-positive test. Subgroup analyses were performed and stratified by major curve location as well as bracing history.

The Pearson correlation coefficient was used to evaluate the relationship between the MCM measurements obtained by X-ray and the App. The intraclass correlation coefficient (ICC) was used to assess the App intra- and inter-rater reliability. Per Fleiss Criteria, correlation coefficients of 0–0.39 are interpreted at poor reliability, 0.4–0.74 modest reliability, and 0.75–1 excellent reliability [12]. Statistical analysis was performed with the R statistical software version 4.3.

## Results

### Demographics

In total, 55 patients were evaluated with a mean age of 13.6 ± 2.1 years (range 10, 18); most patients were female (64%) and white (62%) (Table 1). The average body mass index (BMI) was 19.5 ± 3.6 kg/m2 (range 13.2, 31.8 kg/m2). The mean MCM angle was 21.5 ± 12.5° (range 0, 50). Of these patients, 14 had a larger lumbar curve (mean curve magnitude 18.4 ± 8.2° (range 5, 30)) and 27 had a larger thoracic curve (mean curve magnitude 20.0 ± 9.8° (range 7, 41)). The mean curve magnitude angle for closed triradiate cartilage (*n* = 9) was 21.8 ± 8.0°, closing triradiate (*n* = 2) was 11.5 ± 3.5°, and open triradiate (*n* = 9) was 19.8 ± 10.8°. The correlation coefficient between the MCM measurements obtained by X-ray and the App was 0.804, indicating a strong correlation. In total, 51% (*n* = 28) of patients had a coronal MCM ≥ 20° (Table 1).

**Table 1:** Demographics and clinical characteristics (*n* = 55)

Characteristic	Value
Age (years), mean (SD)	13.6 (2.1) (range 10, 18)
Female, *n* (%)	35 (64)
Race, *n* (%)	
White	33 (60)
Asian	11 (20)
Black	7 (13)
Hispanic	4 (7)
Clinically Significant Scoliosis, *n* (%)	
Yes (major curve magnitude angle ≥ 20°)	28 (51)
No (major curve magnitude angle < 20°)	27 (49)
Major curve magnitude, Mean (SD)	
Angle ≥ 20°	31 (9) (range 20, 41)
Angle < 20°	11 (6) (range 0, 19)
Curvature, *n* (%)	
Primary lumbar curve	14 (25)
Primary thoracic curve	29 (53)
Unknown^1^	12 (22)
Brace, *n* (%)	
Yes	15 (27)
No	31 (56)
Unknown^1^	9 (17)

*SD* standard deviation

^1^Unknown due to limited access to medical record

### Diagnostic accuracy

**Fig. 2:**
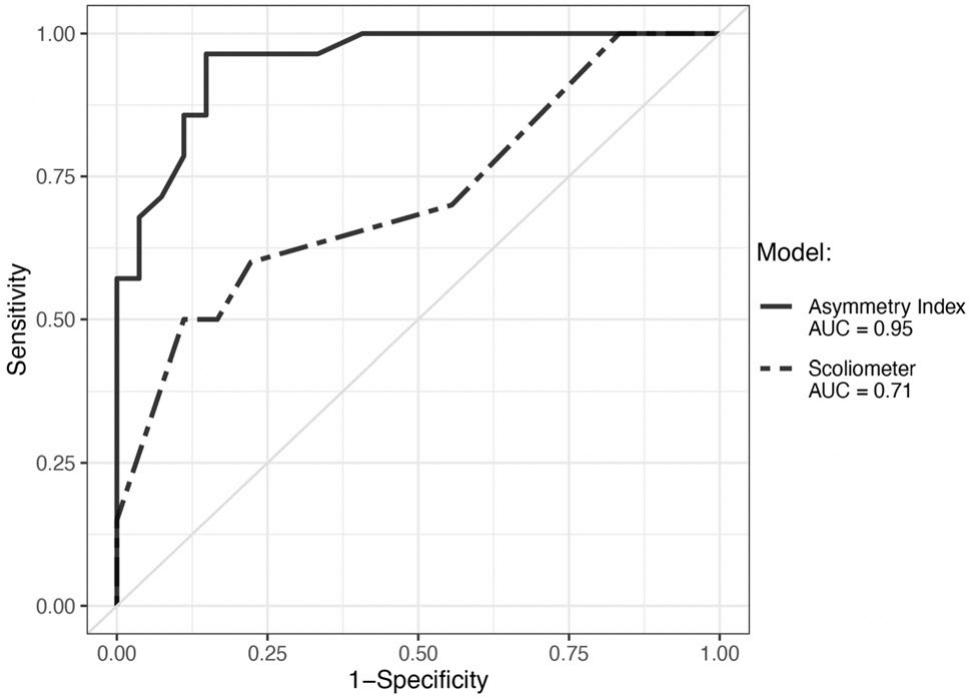
Diagnostic accuracy (ROC AUC) of Scoliosis Assessment App Asymmetry Index compared to scoliometer*. Notes: The area under the ROC curve (AUC) represents an index of diagnostic accuracy with respect to the ability to determine if a patient has a major curve magnitude of ≥ 20. *P = 0.015. *P* value calculated using Delong’s test of two correlated ROC curves. *ROC* receiver operating characteristic

Mean scoliometer measurement of ATR was 8.2 in the MCM ≥ 20° group and 6.2 in the MCM < 20° group. Mean calculated Asymmetry Index was 71.3% in the MCM ≥ 20° group and 42.3% in the MCM < 20° group. ROC analysis indicated larger AUC for Asymmetry Index versus Scoliometer-generated ATR (0.95 versus 0.71; *P* = 0.015; Fig. 2). Using a predictive probability cutoff of 50%, the sensitivity and specificity of the App for diagnosing MCM ≥ 20° was 96.4% (89.6–100% CI) and 85.2% (71.8–98.9% 95% CI), respectively. Using a cutoff ATR of 9.5° the sensitivity and specificity of the scoliometer for diagnosing MCM ≥ 20° was 50% (28.1–71.9% CI) and 88.9% (74.4–100% 95%CI), respectively (Table 2). Overall, the App accurately classified 91% (50/55) of the patients in our dataset with four false positives and one false negative (Table 3). The scoliometer accurately classified 69% (38/55). The results from the subgroup analyses were aligned with the overall results (data not shown).

**Table 2:** ROC AUC, sensitivity, and specificity of Scoliosis Assessment App Asymmetry Index compared to scoliometer

	Asymmetry index		Scoliometer		*P* value
	Estimate	95% CI	Estimate	95% CI	
AUC	0.95	0.90–0.99	0.71	0.55–0.88	0.015
Sensitivity	96.4%	89.6%–100%	50.0%	28.1%–71.9%	< 0.05
Specificity	85.2%	71.8%–98.9%	88.9%	74.4%–100%	NS

*NS* non-significant, *ROC AUC* area under the receiver operating characteristic curve

**Table 3:** Scoliosis assessment app false positives and false negatives*

False positives and false hnegatives						
Age (years)	BMI	Gender	Race	X-ray major curve magnitude (degrees)	App predicted probability (%)	FP or FN
11	13.2	F	White	9	54.10	FP
15	21	M	Black	15	55.30	FP
16	31	M	White	18	61.40	FP
13	19.7	F	Asian	21	43.00	FN
14	22.9	F	Hispanic	19	66.50	FP

A false positive App predicted probability indicated a ≥ 50% chance of clinically significant scoliosis for X-ray major curve magnitude < 20°; whereas a false negative App predicted probability indicated a < 50% chance of clinically significant scoliosis for X-ray major curve magnitude ≥ 20°

*BMI* body mass index, *F* female, *FN* false negative, *FP* false positive, *M* male

^*^X-ray major curve magnitude as “ground truth”.

### Reliability testing

Inter-rater reliability for the App indicated a P value of 0.999 between different rater measurements with ICC of 0.947, which met the criteria for excellent per Fleiss Criteria. The intra-rater reliability ICC score was calculated to be 0.865, also meeting excellent criteria. The inter-rater reliability of personnel completing manual scan alignment had an ICC score of 0.914 which met excellent criteria.

## Discussion

Here we demonstrate the clinical validity (diagnostic accuracy and reliability) of a mobile device-based app to detect clinically significant scoliosis (MCM ≥ 20°) in patients referred for evaluation or ongoing management of scoliosis. Indirect evidence on clinical utility rests on clinical validity. As the evidence demonstrated accurate ST scan test performance, inferences can be made about clinical utility (clinical usefulness). The chain of evidence demonstrates that the Scoliosis Assessment App ST scan can identify individuals with clinically significant scoliosis or progressing disease who would not otherwise be identified using scoliometer or without X-ray radiation; that treatments are available for these patients that would not otherwise be given to patients with clinically significant scoliosis or progressing disease; and that these treatments improve health outcomes. Therefore, a chain of evidence can be created for the Scoliosis Assessment App clinical utility. Possible applications include use in primary care and/or underserved settings as a replacement for scoliometers and alternative to radiographs.

Côté P et al., previously reported scoliometer sensitivity and specificity of 51% and 83%, respectively [4], which is consistent with our scoliometer results (sensitivity 50% and specificity 89%). While cutoffs can be adjusted to maximize sensitivity or specificity according to the intended usage of a test, regardless of the cutoff chosen the App demonstrated significantly higher ROC AUC compared to the scoliometer (0.95 versus 0.71; *P* = 0.015), suggesting that the App could represent a significant improvement over existing diagnostic tools to inform clinical decision making and patient management. Furthermore, we show that inter and intra-rater reliability of the method of obtaining scans is excellent.

In this study, of the 5 patients that had a false positive or false negative result using the App, 4 had a MCM between 15° and 25°. Given that measurement accuracy of coronal MCM angle on a radiograph is ± 5°, these patients are at the border of the diagnostic ability of radiography [13]. This result suggests that in this population the diagnostic accuracy of the App is approaching that of radiographic measurements.

Subgroup analysis demonstrated higher sensitivity and lower specificity for the App among lumbar curves. This is unexpected as prior work has demonstrated lower sensitivity for scoliometer and ST measurements among lumbar curves, perhaps because the ribcage more directly translates rotational spinal deformity into back surface deformity than lumbar paraspinal musculature. Further study is indicated to better explain this finding. We also demonstrated overall improved diagnostic accuracy in unbraced patients (compared to braced), despite those patients having lower curve magnitudes (*P* < 0.001). This may indicate that brace usage may affect body ST independent of its effect on the MCM, potentially altering the relationship of back surface shape to curve magnitude with ongoing brace usage. Further study in the bracing population is indicated to evaluate this effect due to the limited sample size.

Several studies in the past have attempted to predict MCMs utilizing surface topography based approaches. Thometz et al. evaluated the Quantec Spinal Imaging System which calculates MCM from raster stereography and found a statistically significant difference between predicted MCM versus gold standard radiograph of 5.7 ± 9.1° in 237 patients [14]. The Quantec system effectiveness decreased as scoliosis severity increased. Unlike the Quantec system, effectiveness of the Scoliosis Assessment App did not decrease as scoliosis severity increased (that is, more false negatives were not observed with larger MCMs).

Different authors have attempted to improve these results by utilizing varying formulas and landmarks to calculate MCMs. Tabard-Fougere evaluated this same technology in a cohort of 36 patients and found non-significant differences between raster stereography predicted and radiographically measured MCM (*P* = 0.60) [15]. These results had ICC scores of greater 0.75 for both intra-rater and inter-rater reliability. In comparison, the Scoliosis Assessment App achieved inter and intra-reliability measures of 0.947 and 0.865, respectively. Given that the App only requires a smartphone to download and operate, patient access to scoliosis evaluation could be improved compared to alternative technologies.

ST has been utilized to evaluate patients with scoliosis in different applications. Patel et al. analyzed use of 3D contouring to predict scoliosis progression and, in a sample of 38 patients, accounted for 86.9% of variability in future MCM [16]. In comparison to the current study, the authors utilized ST to predict progression as a proof of concept and did not assess the ability of ST to detect clinically significant scoliosis. Komeili et al. utilized ST to evaluate external deformity in accordance with different Lenke classification in 46 scoliosis patients [17]. The study achieved intra and multi observer reliability measures of 0.85 and 0.62. The study did not assess a quantitative measure of asymmetry, and while we had a similar intraclass coefficient (0.865), our results indicated a higher interobserver reliability of 0.947. Frerich et al. evaluated their use of Formetric 4D technology in a study of 64 patients and found a standard deviation of ± 3.4° for measuring MCMs, which falls within the accepted error of 5°.[18] Their results indicated a strong correlation (> 0.70) between radiographic and ST measurements which further supports our correlation (0.804) and the proposed use of ST to diagnose and monitor for clinically significant scoliosis. Lastly, Aulisa et al. compared ST to X-ray and, based on strong ICC scores, concluded that ST can be used to inform diagnosis and treatment including evaluating the curve evolution. [19]

### Limitations

Several limitations are noteworthy. First, despite the limited sample size of 55 patients, the Scoliosis Assessment App demonstrated comparable accuracy relative to X-ray, suggesting that the App may be used as a replacement for scoliometer without diminishing the standard of care. Second, the ST scan and scoliometer test were performed within a 30-day window of X-ray. While scoliosis is unlikely to have meaningfully progressed or improved in 30 days, the 30-day period between X-ray acquisition and acquisition of both the ST scan and scoliometer could have resulted in false-positives or false-negatives. That said, the delay would have similarly affected both the ST scan and scoliometer test. Lastly, the present study was conducted in adolescents, in whom the deformity of the spine from the scoliosis results in visible and measurable changes to the surface topography of the back. In adult scoliosis patients, the pathophysiology is identical, in that the scoliosis results in a twisting of the spine, such that the deformity is similarly able to be identified on the surface of the back [20, 21], suggesting that the results of the present study can be applied to other patient populations and practice settings.

## Conclusion

The Scoliosis Assessment App using surface topography technology offers an accurate, scalable, and radiation-free method of detecting and monitoring patients for clinically significant scoliosis, with comparable accuracy relative to X-ray and significantly improved performance compared to scoliometer. Future research will focus on the use of the App in expanded patient populations as well as longitudinally.

## Data Availability

The data presented in the study are included in the article. Any further data inquiries can be directed to the corresponding authors.
